# Electrospun Functional Materials toward Food Packaging Applications: A Review

**DOI:** 10.3390/nano10010150

**Published:** 2020-01-15

**Authors:** Luying Zhao, Gaigai Duan, Guoying Zhang, Haoqi Yang, Shuijian He, Shaohua Jiang

**Affiliations:** 1 Co-Innovation Center of Efficient Processing and Utilization of Forest Resources, College of Materials Science and Engineering, Nanjing Forestry University, Nanjing 210037, China; zhaoluying1@163.com (L.Z.); shuijianhe@njfu.edu.cn (S.H.); 2 College of Chemistry and Molecular Engineering, Qingdao University of Science and Technology, Qingdao 266000, China; zhanggy@qust.edu.cn; 3 College of Material Science and Engineering, Jilin University, Changchun 130022, China

**Keywords:** electrospinning, food packaging, functional membrane, nanofibers

## Abstract

Electrospinning is an effective and versatile method to prepare continuous polymer nanofibers and nonwovens that exhibit excellent properties such as high molecular orientation, high porosity and large specific surface area. Benefitting from these outstanding and intriguing features, electrospun nanofibers have been employed as a promising candidate for the fabrication of food packaging materials. Actually, the electrospun nanofibers used in food packaging must possess biocompatibility and low toxicity. In addition, in order to maintain the quality of food and extend its shelf life, food packaging materials also need to have certain functionality. Herein, in this timely review, functional materials produced from electrospinning toward food packaging are highlighted. At first, various strategies for the preparation of polymer electrospun fiber are introduced, then the characteristics of different packaging films and their successful applications in food packaging are summarized, including degradable materials, superhydrophobic materials, edible materials, antibacterial materials and high barrier materials. Finally, the future perspective and key challenges of polymer electrospun nanofibers for food packaging are also discussed. Hopefully, this review would provide a fundamental insight into the development of electrospun functional materials with high performance for food packaging.

## 1. Introduction

Electrospinning is a versatile technique for continuously producing nanofibers with a fiber diameter range from sub-nanometers to micrometers. The electrospun fibers have been broadly applied in nearly all the fields, such as composites [1,2,3,4,5], tissue engineering [6,7,8,9], biomaterials [10,11], energy storage and conversion [12,13,14,15,16], food packaging [17,18,19], drug deliver and release [20,21], catalysts [22,23,24,25], sensors [26,27,28,29], flexible electronics [30,31,32], reactors [33,34], environmental protection [35,36,37], etc. During the fiber preparation process, the polymer solution or melt is induced by a high-voltage power supply device to accelerate injection onto a collecting plate with opposite polarity to form nanofiber membrane. Basically, there are three key components to fulfill the process: a high voltage supplier, a pipette or needle with small diameter, and a metal collector [38]. In details, during the electrospinning process, the polymer solution is extruded from the capillary tube by the electric field force, and a Taylor cone can be formed at the tip of the capillary. As the strength of electric field increases, positive charges could accumulate on the surface of the Taylor cone, which further overcomes the surface tension and cause fluid ejection. When the spinning process proceeds, the injected fluid could be stretched several times longer than the original length, and the solvent evaporates simultaneously to form a continuous ultrafine polymer fiber.

The electrospinning process is a simple and effective strategy for fabricating nanofibers, which can prepare polymer nanofibers directly, continuously and even in a large scale. It has the advantages of mild experimental conditions, low cost, easy operation and function, wide range of raw materials, etc. The spinning process is controllable, and the parameters can be adjusted according to the different requirements in various research fields. For example, electrospun nanofibers can be prepared with custom shapes and various orientations to quantitatively investigate the relationship of mechanical properties and molecular orientation [39]. Generally, the nanofibers obtained by electrospinning would have the characteristics of fine size, large specific surface area, high porosity, large aspect ratio and superior mechanical properties. 

**Figure 1:**
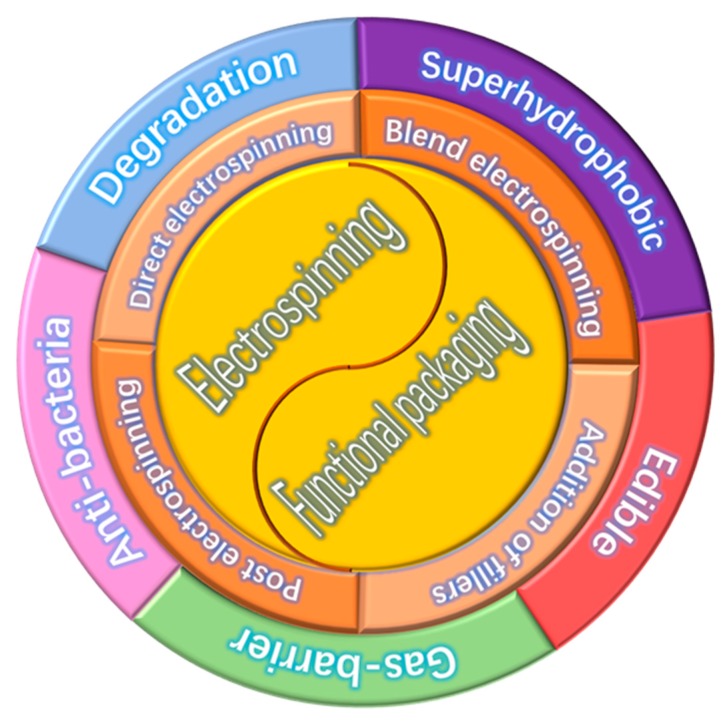
Overview of functional electrospun and food packaging materials diagram.

Functional packaging materials are gradually evolving into the public eyes, which have the functions of moisture absorbing [40,41], antioxidant releasing [42,43] and flavor or odor absorbing [44]. However, for the functional packaging materials applied in the food field, some additional features must be considered, such as degradable, superhydrophobic, edible, antibacterial and high barrier. By virtue of their submicron to nano-scale diameter and very large surface area, electrospun fibers may offer numerous advantages compared to conventional film and sheet packages, such as being more responsive to changes (e.g., relative humidity and temperature) in the surrounding atmosphere. Furthermore, because the electrospinning process takes place at ambient conditions, electrospun fibers are more suitable for encapsulating thermally labile active agents as compared to the fibers made by conventional melt spinning process. Given these advantages mentioned above, electrospun fiber not only could incorporate into bioactive substances, but also could satisfy the requirements of designers and consumers for packaging materials. Therefore, the development of functional packaging materials based on electrospinning technology has become a hot spot in the food packaging field. In the following sections, different approaches for the preparation of functional electrospun fiber will be introduced and their applications on functional package materials will be described (Figure 1). Furthermore, a conclusion including future perspective and key challenges for electrospun functional packaging materials are also discussed. In a word, we believe electrospun materials are good candidates for food packaging materials, and this review would significantly promote the research on application of electrospun fibrous materials for food packaging materials.

## 2. Strategies for the Preparation of Functional Electrospun Materials

In 1934, Formalas invented an experimental device for preparing polymer fibers by electrostatic force and applied for a patent that discloses how a polymer solution forms a jet between two electrodes. The above device could successfully produce a fiber by using high voltage static electricity, which is consequently recognized as the beginning of electrospinning technology [45]. Unfortunately, electrospinning technology did not attract numerous attentions until the middle of the 20th century. With the rapid development of nanomaterials and nanotechnology, electrospinning method has gradually received the attention of scholars from various areas. 

So far, the preparation method for nanofibers based on electrospinning technology has been well developed. According to the electrospinning raw materials, it can be divided into melt electrospinning [46,47,48], solution electrospinning [49,50] and mixed electrospinning [51,52,53]. According to the design of the spray head, it can be classified as needleless electrospinning [54,55,56], coaxial or triaxial electrospinning [57,58,59,60,61,62], multi-jet electrospinning [63,64,65], etc. The application of electrospinning technology will be described below according to different situations.

### 2.1. Direct Electrospun Packaging Membrane

Direct electrospinning is defined here as single-component melt electrospinning or single-component solution electrospinning using one jet head. The functional electrospun materials commonly used in packaging field are chitosan (antibacterial), corn protein (edible), polyvinyl alcohol (transparent), etc.

Chitosan (CS) is obtained by deacetylation of chitin, which could form a transparent, elastic and oxygen resistant film. CS film can not only prevent fungi from contaminating and corroding food, but also effectively regulate the composition of oxygen and carbon dioxide around fruits and vegetables, inhibiting the aerobic respiration to a certain extent, so as to improve the shelf life. CS has a huge application potential in the food industry attribute to its advantages of short-time biodegradation, biocompatibility with human tissues, anti-microbial and antifungal activities and non-toxicity. Therefore, chitosan-based nanofiber membrane/film has attracted great attention in food preservation and packaging technology [66,67].

Ohkawa et al. [68] successfully prepared pure CS electrospun nanofibers with trifluoroacetic acid (TFA) as spinning solvent for the first time, because TFA can form salt with amino group in chitosan, effectively reducing the interaction between CS molecules, making electrospinning easier. In addition, the high volatility of TFA is beneficial to the rapid solidification of CS-TFA electrostatic jet. The concentration of CS also affects the morphology of fibers. When the mass fraction of chitosan is 6% or lower, beads and fibers coexist. When the mass fraction of CS is 7%, beads obviously decrease. When the mass fraction of CS is 8%, the spinning effect is better. The diameter of obtained fibers ranges from 390 to 610 nm, with an average diameter of 490 nm, but there still are small beads and interconnected fibers can be seen. To avoid the above phenomena of beads and interconnected fibers and improve the uniformity of electrospun fibers, dichloromethane (DCM) was added into chitosan-TFA solution. Under the optimum conditions, uniform CS nanofibers with an average diameter of 330 nm can be obtained. 

In addition to the TFA as solvent, another effective solvent for chitosan is concentrated acetic acid. Geng et al. [69] studied the electrospinning of CS with concentrated acetic acid as solvent. The results show that with the increase of acetic acid concentration, the surface tension of chitosan-acetic acid solution decreases, while the viscosity does not change significantly. At the same time, the charge density of the jet increases. When the mass fraction of acetic acid is 30%, nanofibers begin to appear, accompanied with a large number of beads; when the mass fraction is 90%, uniform fibers with an average diameter of 130 nm can be obtained, and no beads appear. The molecular weight and concentration of CS also affect the formation of fibers. Only when the molecular weight of CS is about 1.06 × 10^5^ g/mol and the mass fraction is 7–7.5%, the beadless nanofibers can be produced. However, high content of CS (more than 90%) cannot be well dissolved in solvent, which is difficult to meet the requirements of spinning viscosity. In addition, the electric field strength also affects the formation of fibers. When the electric field strength is 1 kV/cm, spindle beads and coarse fibers appear. When the electric field strength is 3–4.5 kV/cm, uniform and regular fibers can be formed. However, when the electric field strength is greater than 4.5 kV/cm, a great number of beads could exist in the obtained fine fibers due to the tensile force increases and the charged jet is unstable.

Besides the solvent composition and concentrate, the fiber morphology is also influenced by other parameters, such as applied voltage, needle diameter, receiving distance and feed rate. For instance, Sencadasa et al. [70] investigated the effects of electrospinning parameters on the average diameter and width distribution of CS nanofibers. By controlling the solution parameters and process parameters, the spinning process gets more stable, and the target fiber size could be customized. However, the parameters of the needle tube diameter and feeding speed have no effect on the fiber diameter. When the applied voltage is increased, the diameter of the fibers would decrease. Furthermore, with the reduction of receiving distance, the fibers diameter at the tip of the needle decreases due to the decrease of electrostatic field intensity.

Zein, a kind of storage protein in corn, has a good film-forming property owing to its rich sulfur-containing amino acids that could connect the disulfide bond and water release bond. As a renewable polymer protein, zein has good biocompatibility, which can be used not only as fresh-keeping film, but also as film coating for food preservation [71]. Moreover, it can also be used as edible packaging films in food packaging.

Takanori et al. [72] investigated the parameters such as electric field and polymer solution concentration in the electrospinning process to prepare zein nanofiber membranes. They choose 15 kV and 30 kV as the field strength and 80 wt.% ethanol aqueous solution as solvent. It is found that when the electric field strength is 15 kV, the polymer concentration is mainly 21 wt.%. However, the electric field strength is increased to 30 KV, fibers could be generated at 18 wt.% of polymer concentration. Neo et al. [73] also prepared the electrospun zein membranes with the 80% ethanol as solvent, and further studied the fiber morphology affected by three processing parameters including solution concentration, electric field strength and solution parameters. That is, with the increase of zein concentration, the viscosity of solution would increase, which promotes the formation of bead-free fiber, and the average fiber diameter become larger. They also found that there is an interaction effect between the electric field and feed rate of the solution. At a high voltage, changes of the fiber diameter have a more sensitive response to the feed rate of the solution. Similarly, changes in fiber diameter were also found to be more responsive to the applied voltage at a high feed rate. However, at a low applied voltage, the effect for the feed rate on average fiber diameter will not be significant since the amount of charges are not enough to accelerate the solution.

Polyvinyl alcohol (PVA) film is a kind of antistatic film with good performance, which is widely used in the sales and packaging of textiles and clothing. Compared with polyethylene, polypropylene and other general-purpose films, polyvinyl alcohol film exhibits the advantages of high transparency, good antistatic property, which can significantly reduce the dust absorption effect [74,75,76]. Furthermore, polyvinyl alcohol is often used in water-soluble packaging.

**Figure 2:**
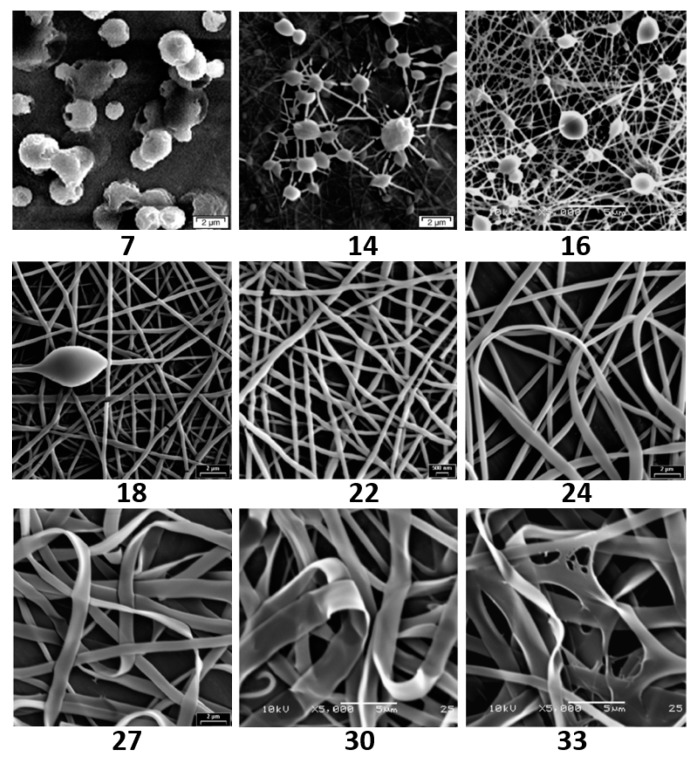
Photographs showing the range of structures that can be produced as the concentration (wt.%) is increased at Mw = 18,000 g/mol [77]. © 2006 Elsevier B.V. All rights reserved.

Tao et al. [77] studied the effects of molecular weight and concentration in a PVA-water system on the morphology and structure of PVA electrospun nanofibers (Figure 2). They found that with the increase of molecular weight, the morphology of PVA fibers can be changed from bead to beaded fibers, then to smooth fibers and finally to flat ribbons at the same concentration. Supaphol et al. [78] further explored the effects of solution concentration and parameters change during electrospinning process (applied voltage and collection distance) on the morphology and fiber diameter of as-spun PVA fiber mats and individual fibers. In the range of solution concentration (6–14%), the average fiber diameter for as-spun PVA fiber mats decreases with the increase of solution concentration and applied voltage, which ranges from 12.5 to 25 kV. At a fixed applied potential of 15 kV, when the receiving distance was changed within a certain range (5–20 cm), the average fiber diameter would become smaller as the receiving distance increased. In addition, there is a decrease of the viscosity that can be observed after sonication, which could result in a decrease of average fiber diameter.

In another study, Yang et al. [79] employed bubble electrospinning, a special electrospinning method, to prepare PVA nanofibers, and studied the effects of solution concentration and viscosity on the morphology and fiber diameter. They concluded that the higher PVA concentration in solution, the smoother surfaces and larger diameters in the obtained nanofibers. There is an allometric scaling relation between the diameter and PVA aqueous solution concentration: *d∝C^5*. Niu et al. [80] fabricated PVA nanofibers via a needless method, which utilized a cone-shaped metal wire coil as a spinneret instead of needle. This method would increase the fiber production efficiency and achieve a finer average fiber diameter than that of traditional electrospinning. In the same year, this group [81] reported a needleless electrospinning method including a rotating disk and cylinder to prepare PVA nanofibers, and discussed the effect of nozzle shape on spinning process and fiber morphology. On one side, this needleless electrospinning method could improve the productivity, which overcomes the shortcomings of the traditional multi-needle electrospinning method such as large operating space and careful design. On the other side, needleless electrospinning avoids strong charge repulsion between the spinneret and adjacent needles, resulting in an even fiber deposition.

Apart from three polymers mentioned above, some degradable polymers have also been applied in food packaging field, such as polycaprolactone (PCL), poly (propylene carbonate) (PPC), polylactic acid (PLA) and natural cellulose, starch. Moreover, electrospinning plastic materials, such as polyvinyl chloride (PVC), polyethylene (PE), polypropylene (PP), polystyrene (PS), polyethylene terephthalate (PET) and nylon, are commonly used in the field of packaging as well.

### 2.2. Mixed Electrospun Packaging Membrane 

Electrospinning of polymer blends enables the produced nanofibers possess multi-function from each constituent. Up to now, there are many ways for solution mixing, such as electrospinning of multi-components mixed solutions, and design of jet device to obtain multi-components mixed fiber membranes. In the food packaging industry, electrostatic spinning technology is usually employed to prepare composite mats of two or more components.

#### 2.2.1. Blending with Different Polymer Solutions

Before electrospinning, by mixing two or three polymers in spinning solutions, the fiber membranes with better performance could be obtained. Recently, some natural and natural-derived polymers have been used to prepare electrospun fibers through blending with other functional polymers in spinning solutions.

Feng et al. [82] blended gelatin (GT) and PCL (50:50) in trifluoroethanol (TFE) cosolvent, and found a phase-separation behavior of the GT/PCL polymers, which leads to inferior morphology of fibers during the electrospinning. In order to solve this problem, they added a tiny amount of acetic acid to the mixed solution. As a result, the opaque solution became transparent immediately without any obvious precipitation for over one week, and the obtained nanofibers demonstrated a thin, smooth and homogeneous morphology.

Chitosan is soluble in many solvents. It is soluble in organic acids, such as dilute acetic acid, formic acid and lactic acid, and also soluble in the mixture of water and methanol, ethanol or acetone, and its physical and chemical properties can be used for electrospinning, so it is often mixed with other polymers to prepare electrospinning fibers. González et al. [83] studied the thermophysical properties of CS + starch + PET fibers via a blending electrospinning, which could have excellent mechanical properties, thermal stability and biodegradability due to the strong intermolecular force among three components verified by Infrared spectra. Sajeev et al. [84] electrospun PVA and CS in a mixed homogeneous solution to prepare nanofibers and fiber mats. The effects of parameters (such as flow rate, receiving distance and voltage) on the morphology and fibers diameter were also investigated. It was found that the effects of parameters on fibers accorded with a simple extensional creep model. In addition, there is a negative correlation between the content of CS and fibers diameter, which is because the CS is an ionic polyelectrolyte that could enhance the charge density of the surface of jet stream and the received electric field force, causing a decrease of fiber diameter. 

#### 2.2.2. Blending by Multiple-Jet Electrospinning

**Figure 3:**
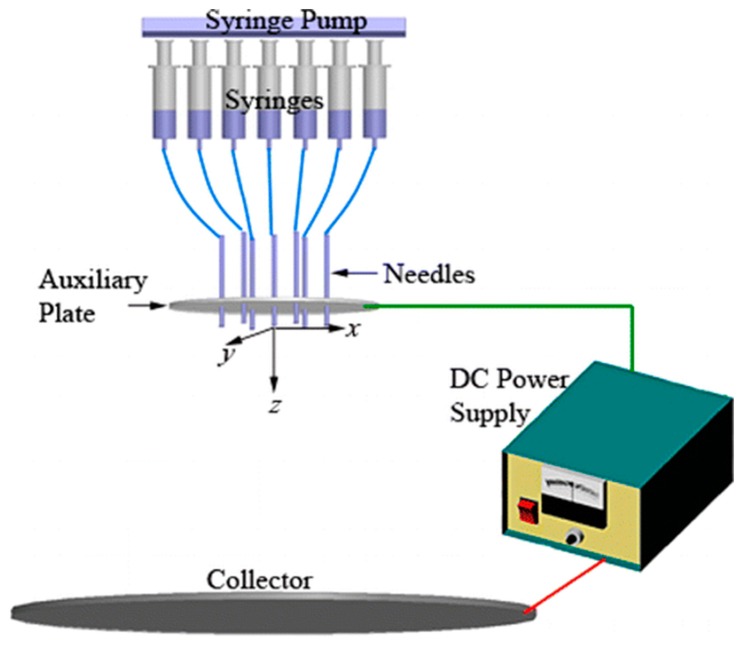
A schematic of the experimental setup used in the multi-jet electrospinning process [85]. © American Chemical Society 2014.

Traditional single-nozzle electrospinning method has restricted its industrial application because of its low output. After years of research and design, the combination of multiple nozzles with different specifications and quantities has been developed.Different nozzles are injected with various solutions, which are mixed and entangled into fibers, then deposited into mats during the electrospinning (as shown in Figure 3, [85]). For example, Ding et al. [86] prepared a series of biodegradable PVA/cellulose acetate (CA) nanofibrous mats via a multi-jet electrospinning method. The weight ratio of the PVA and CA in fiber mats can be controlled by changing the number ratio of PVA/CA jets. Moreover, this multiple-jet electrospinning process ensures a good dispersion of two polymers in the PVA/CA hybrid felts.

**Figure 4:**
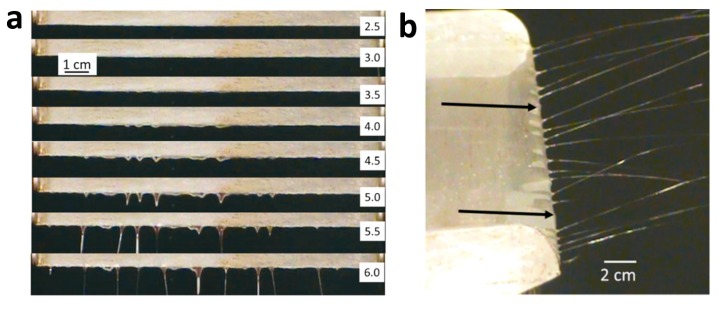
(**a**) Sequential images of the polymer-melt coated plate edge showing the progression of the fluid with time (in minutes, right side) at 180 °C at an applied voltage of −45 kV. The plate is oriented horizontally and being viewed from above. (**b**) Image of electrospinning from a polymer melt coated source plate (PE at 170 °C, −45 kV) in steady state (i.e., after 30 min). The black arrows indicate non-jetting perturbations. This figure is adapted from [87]. © 2014 IOP Publishing Ltd.

The method described below can also be used to prepare hybrid electrospun fibers. Wang et al. [87] utilized a special multi-jet electrospinning method to spin the melted polyethylene and PCL, which actually was an unconfined spinning geometry instead of a needle as performed in Figure 4. In this process, due to the influence of the applied electric field, numerous parallel jets of molten polymer are formed at the plate edge, and finally polymer films are obtained on the collector. Compared with traditional needle electrospinning, unconfined geometries [88,89,90,91] rely on electric-field induced spontaneous fluid perturbations to form jet sites, rather than mechanically pumping fluid through a confining nozzle. As the nozzle blockage does not occur in this device, and nanofibers from parallel jets could be prepared synchronously in such an open configuration, so that the production rate has been greatly improved. It is a convenient and high-throughput method for industrial production of microfibers and nanofibers with thermoplastic or other high-viscosity fluids.

#### 2.2.3. Blending by Coaxial Electrospinning

Coaxial electrospinning could produce a new type of nanofibers with a core-shell structure. In the coaxial electrospinning [57], the syringe is designed as two chambers, the outside chamber is generally filled with the polymer solution, and another polymer solution or powders in the inside chamber. During the electrospinning, a core-shell droplet firstly appears at the outlet of the core-shell needle, and then forms the core-shell structure fibers under the force of electric field.

Yao et al. utilized coaxial electrospinning technology to successfully prepare a novel antibacterial composite through encapsulating orange essential oil (OEO) in zein prolamine. The obtained fine fibers ranging in diameter from 0.7 to 2.3 μm depending on the zein prolamine solution concentration and process parameters. Fiber composites exhibit an antibacterial activity against *Escherichia coli* and can be used as food packaging materials for bioactive food preservation, such as extending the shelf life of fruits [92].

**Figure 5:**
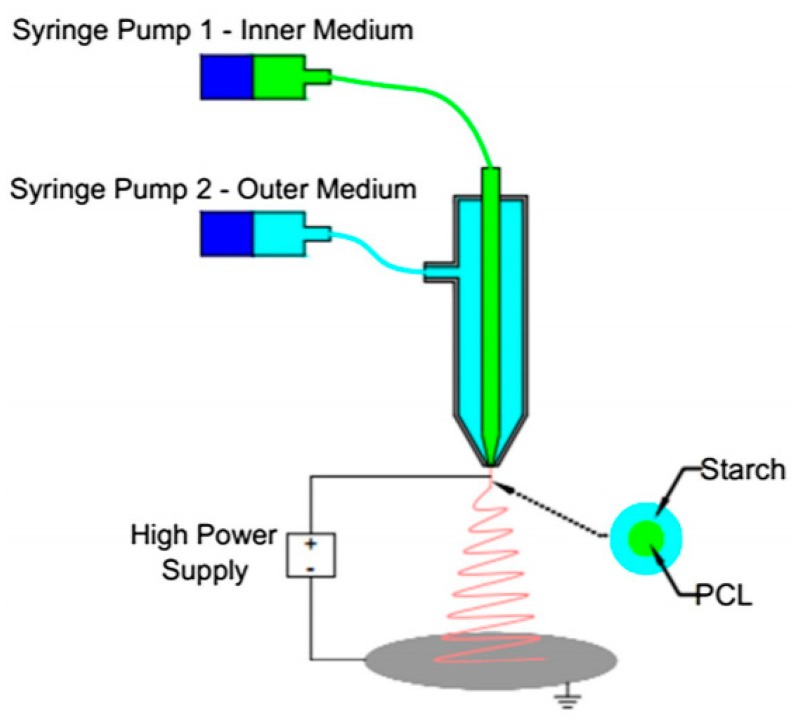
Schematic representation of the coaxial needle electrospinning set-up [94]. © The Korean Society of Pharmaceutical Sciences and Technology 2012.

It is common that the shell and core are different polymers. Komur et al. [93] fabricated starch and PCL composite nanofibers by coaxial needle electrospinning technique as shown in Figure 5. In this paper, the PCL solution was fed through the inner needle, and the starch solution was located at the outer medium. It was found that processing parameters have a significant influence on the fibers diameter, including polymer concentration, flow rate and voltage. The effect of starch ratio on the physical properties and morphological structures of fiber was also studied. That is, the higher the proportion of starch, the greater of the electrical conductivity, viscosity and density, while the beads would more likely appear in the fibers due to the starch tends to prefer to form beads rather than fibers. When the ratio of the starch increases, the volume of beads would become larger, which will cause the average fibers diameter becomes larger and the fibers strength gets higher. Park et al. [94] prepared levofloxacin-loaded CS and PCL nanofibers by coaxial electrospinning to control the release of antibiotics. They employed CS containing levofloxacin as a core, and PCL as a shell. PCL with different concentrations (8, 12, 16 and 20 wt.%) were set up to explore the effects of nozzle shapes on the sustained release of levofloxacin. As expected, the CS-PCL nanofiber scaffolds with coaxial nozzles had better performance on the sustained release of levofloxacin. Alharbi et al. [95] prepared PLA/PVA and PVA/PLA nanofibers with core/shell-structure to study the mechanical properties of PLA and PVA composite. According to the mechanical test results under static loading, the tensile strength and plasticity of core/shell PLA/PVA nanofibers increased by nearly 233% and 150% respectively compared with the original value. Dynamic loading and creep loading experiments show that there is a strong physical interaction between PLA layer and PVA layer, which could improve the mechanical properties.

**Figure 6:**
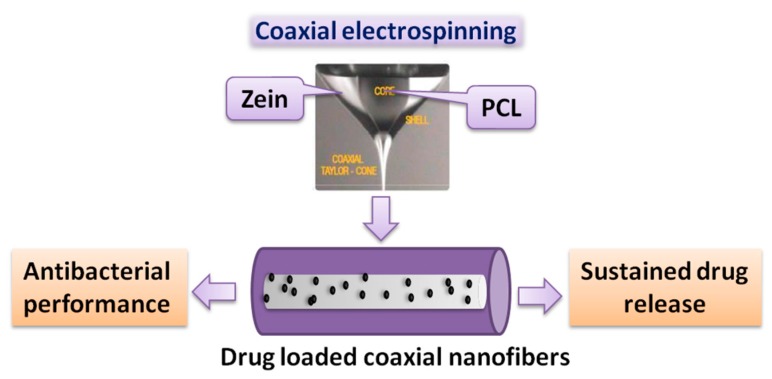
Structure diagram of coaxial fiber [97]. © Elsevier Inc. All rights reserved 2016.

Sometimes, functional substances such as antibacterial substances are loaded into the core of the coaxial electrospun fiber, which would release later so as to be effective. Korehei et al. [96] directly incorporated the T4 bacteriophage into the fiber core to prepare a core/shell fiber structure via a coaxial electrospinning method. The electrospun fibers were produced using a PEO/chloroform solution as the shell and a T4 bacteriophage/buffer suspension as the core. Coaxial electrospinning could produce continuous core/shell fibers with bead-free morphology, and the T4 bacteriophage was uniformly incorporated in the core of fibers. The core/shell fiber encapsulated bacteriophage exhibits full bacteriophage viability after storing for several weeks at +4 °C, which can be applied in meat packaging. He et al. [97] employed coaxial electrospinning technology to prepare anti-infective drug delivery carriers (Figure 6), i.e., poly (e-caprolactone)/zein uniform beadless core/shell nanofibers loaded with metronidazole (MNA). The prepared fiber membranes were hydrophobic and could inhibit the growth of anaerobic bacteria by releasing MNA.

### 2.3. Addition of Inorganic Fillers

In order to achieve multi-function for nanofiber-based food packaging materials, various fillers have been added into polymer substance, especially inorganic fillers that possess conductivity, magnetism, antibacterial property, etc. [98,99,100,101]. Then, a nanofiber-based food packaging material could be prepared through the electrospinning technique.

#### 2.3.1. Conductive Fillers

Electrostatic hazard is a common problem in the packaging industry. Some severe accidents and impacts, such as fire, explosion and dust absorption are related to the electrostatic effect. Generally speaking, the insulating material is easy to produce static electricity. Adding conductive fillers is an efficient way to solve the problem and enhance the antistatic performance. Moreover, conductive packaging materials have a great potential for application in smart packaging due to their combination with some electronic devices.

Carbon-based conductive materials are often used as conductive additives to prepare conductive/anti-static packaging materials with wide application and large usage [102]. Carbon black has been widely adopted, but it has a huge loss of mechanical properties for the composites. By comparison, carbon nanotubes (CNTs) exhibit the advantages of less filling amount and negligible mechanical properties effects, which are emerged as a new generation of carbon-based conductive materials. Yang et al. [103] prepared electrospun biodegradable PLA composites which using CNTs as conductive fillers. They found that the morphology of the fibers is related to the loading of CNTs. At a low loading level, the CNTs can be well embedded in the PLA matrix to form a fiber axis-oriented nanowire structure. At a high loading level, the CNTs are mainly dispersed in the form of bundles along with the fiber axis, and the resulting fibers are tortuous or misshaped. Meng et al. [104] studied the effects of different multiwalled CNTs (MWCNTs) content (0.1%, 0.5%, 1%, 2% and 5%) on the properties of non-bead PCL-MWCNTs nanofiber membranes, which found that the diameter distribution, average diameter, crystallinity and tensile strength are increased with the addition of MWCNTs. Moreover, the electrospun PCL–MWCNTs nanofiber membranes also exhibited a good degradability. Giner et al. [105] obtained electrospun nanocomposite fibers mats by embedding graphene nanoplatelets (GNPs) in poly (ethylene-co-vinyl alcohol) (EVOH) fibers. The heat-treated fibers mats at 158 °C could produce continuous, contact and transparent films, which have a great potential to be applied in the intelligent packaging field. For instance, smart labels or tags can be anticipated.

#### 2.3.2. Magnetic Fillers

For various electronic components, such as electronic precision instruments, medical devices, computers, automatic office equipment and other products, are sensitive to electromagnetic radiation, therefore, the anti-electromagnetic radiation packaging is urgently needed. Adding magnetic filler is an effective way to endow materials with anti-electromagnetic radiation performance. Recently, Iron oxides or compounds have been often employed as the magnetic fillers for electrospun nanofibers.

Song et al. [106] encapsulated self-assembled iron-platinum (FePt) magnetic nanoparticles in PCL nanofibers by coaxial electrospinning. The discrete FePt nanoparticles array can be arranged in a long-range order along the fiber axis at 3000 nm. Wang et al. [107] prepared Fe_3_O_4_/PVA composite nanofibers by combining in-situ polymerization and electrospinning technology. The Fe_3_O_4_ magnetic fluids were synthesized through chemical co-precipitation method in the presence of 6 wt% PVA aqueous solution, which avoids the agglomeration of magnetic nanoparticles due to the stabilizing effect of PVA. Wei et al. [108] prepared a porous magnetic biodegradable Fe_3_O_4_/CS/PVA nanofiber membranes, and explored the influence of electrospinning parameters (polymer concentration, Fe_3_O_4_ content and magnitude of applied voltage) on the morphology of fibers. When the polymer concentration was 4.5 wt.%, the applied voltage was 20 kV and the loading of Fe_3_O_4_ nanoparticles was less than 5 wt.%, the uniform, smooth and continuous Fe_3_O_4_/CS/PVA nanofiber membrane can be obtained. Stylios [109] reported the effect of processing parameters on the morphology of PVA/FeCl_3_ magnetic composite fibers, such as applied voltage, receiving distance, flow rate and solution concentration. This research provides guidance for the subsequent manufacture of flexible magnetic composite membranes. Kumar et al. [110] electrospun the PLA, PEG and magnetic nanoparticles (Fe_3_O_4_@SiO_2_ core-shell NPs) mixed solution at room temperature to prepare a series of fibers. With other parameters remaining unchanged, the fiber diameter was reduced from 6 to 3 micrometers after the PLA solution was added to PEG. When the PLA solution mixed with PEG + MNPs (magnetic nanoparticles), the fiber diameter was decreased from 3 to 1 μm.

#### 2.3.3. Photocatalytic Fillers

Photocatalytic fillers have the ability to impart the self-cleaning properties to functional packaging materials. Many scholars demonstrate the self-cleaning ability of materials by testing the rate of dyes degradation with different colors.

**Figure 7:**
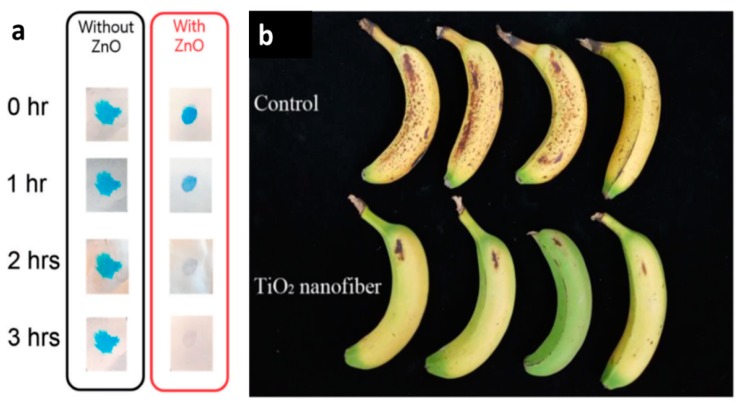
(**a**) Comparison of photocatalytic activity of fibers with and without ZnO [114], © 2018 SAGE Publications. (**b**) photographs of bananas stored for 10 days covered with PP film (control) or PP film and nanofiber containing 5 wt% TiO_2_ [115]. © Springer Science+Business Media, LLC, part of Springer Nature 2018.

TiO_2_ is a non-toxic, low-cost and good photocatalytic filler, which is often used to treat pollutants. Bedford et al. [111] prepared a photocatalytic self-cleaning fiber membrane through a coaxial electrospinning device, which employed a CA solution as a core and the TiO_2_ dispersion as a shell. The obtained self-cleaning fiber membrane had been tested to completely degrade the blue dye within 7–8 h when exposed to a halogen lamp (Figure 7). Nasikhudin et al. [112] prepared PVA/TiO_2_ composite nanofibers by electrospinning method, and studied the photocatalytic activity for the degradation of methylene blue under ultraviolet light. It was found that the PVA/TiO_2_ composite nanofibers suspended in dye solution could degrade 70% of the dye within 5 h.

ZnO is often doped as the filler into photocatalytic composite fibers because of its simple synthesis, non-toxic and pollution-free. Liu et al. [113] electrospun precursor solution of zinc acetate (ZnAc)/CA in mixed-solvent of N, N-dimethylformamide/acetone, then calcined that to obtain photo-catalytically active ZnO nanofibers. According to experiments, they found that ZnO nanofiber mats could degrade nearly 60% of Rhodamine B within 2 h under visible light irradiation. Khan et al. [114] successfully manufactured ZnO/poly (1,4-cyclohexanedimethylene isosorbide terephthalate) (PICT) nanofibers by electrospinning technique. When the load concentration of ZnO is 9% and the concentration of PICT is 10%, the composite fiber can achieve 99% self-cleaning efficiency within three hours exposed to UV light (Figure 7a). Apart from endowing the composite electrospun fiber membrane with self-cleaning ability, some photocatalytic fillers can also remove some organic compounds in the package. For example, Zhu et al. [115] prepared the electrospun PP films, which can be potentially be used as packaging material for bananas, because TiO_2_ loaded in the fiber membrane would catalyze the degradation of ethylene and delay the excessive ripeness and deterioration of banana fruits (Figure 7b).

#### 2.3.4. Antibacterial Fillers

Antimicrobial packaging materials has played a majority role in the field of food packaging, which could limit or prevent the growth of food spoilage bacteria or pathogens by prolonging the stagnation period of microorganisms, slowing down the growth rate or reducing the survival number of microorganisms, so as to ensure the quality and extend the shelf life of food. So far, there are three kinds of common antibacterial agents, which are the inorganic antibacterial agent, organic chemical antibacterial agent and natural biological antibacterial agent, respectively. Inorganic antibacterial agents mainly refer to some metal ions (such as Ag^+^, Cu^2+^, Zn^2+^, etc.) or their compounds that would have antibacterial activity, and also the atomic oxygen sterilization, which is produced by a photochemical reaction. Among them, zinc oxide and titanium dioxide are the most investigated in previous reports. The main characteristics of inorganic antibacterial agents are good heat resistance, wide antibacterial range, long effective antibacterial period and not easy to produce drug resistance [116].

**Figure 8:**
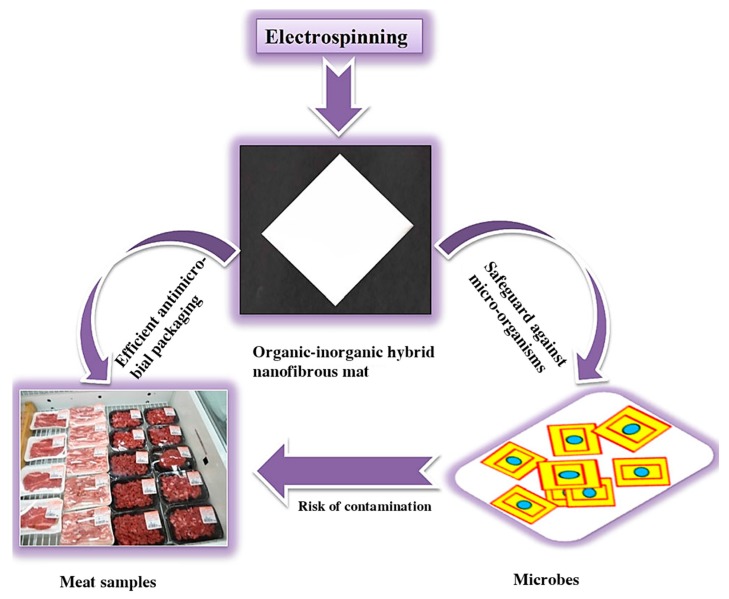
Electrospun hybrid mats; its antimicrobial concept and projected future applications as packaging material for meat and meat-based products [117]. © Association of Food Scientists & Technologists (India) 2014.

Amna et al. [117] reported for the first time the fabrication of olive oil/polyurethane (PU) composite nanofibrous packaging mats decorated with ZnO nanoparticles by electrospinning. PU is thermoplastic polymer that demonstrates outstanding mechanical properties and water insolubility. Meanwhile, PU possesses good barrier properties, oxygen permeability and more like to the FDA (Food and Drug Administration) point. Olive oil, a natural material, is often loaded with various antibacterial ingredients, flavonoids and antioxidants. Likewise, zinc oxide nanoparticles (ZnO) possess antibacterial activity, which is low toxicity and biodegradability. These kinds of biodegradable packaging materials have displayed a potential antimicrobial activity against *S. aureus* and *S. typhimurium*. Therefore, it can be used for packaging fresh or processed meat and meat-based products (Figure 8). The synthesized nanofibers have the opportunity to replace non-degradable films and overcome the complexity of recycling.

### 2.4. Post-Treatments of Electrospinning Membrane

Post-treatments, such as hot working, surface modification, dip coating, etc., could greatly enhance the performance of electrospun membranes, even expand the application range to meet the high requirements of designers.

#### 2.4.1. Thermal Treatments

By heating the electrospun membrane, the nanofibers in the electrospun membrane can be crosslinked, and the properties such as mechanical properties can be further improved. For example, Lee et al. [118] fabricated PVA nanofibers crosslinked with blocked isocyanate prepolymer (BIP) by the electrospinning process and subsequent thermal treatment. Their experiment showed that a chemical cross-linking reaction occurs between the hydroxyl group of PVA and the isocyanate group of BIP during the thermal treatment. After the chemical cross-linking finished, PVA/BIP nanofibers not only have a significant improvement in mechanical properties and water resistance, but also have an increased thermal stability. In addition, the fibers membrane has the general advantages of electrospun nanofibers, which is owing to a large surface area and high porosity, resulting in a wider application potential. Lee et al. [119] employed an electrospinning technology to prepare PCL fibers. In order to improve the mechanical properties, the fibers were placed in Pluronic F127 solution and heated at a various temperature ranging from 54 to 60 °C for 30 min. After the heat treatment, bonding occurs between fibers, and the biomechanical properties are improved significantly. Thus, these fibers possess adequate tensile properties, suture retention strength and burst pressure strength.

**Figure 9:**
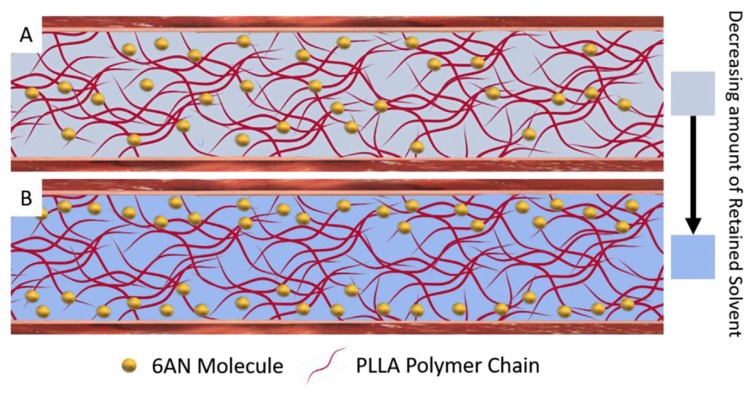
Schematic of the effects of solvent removal on the 6AN distribution inside of an individual electrospun nanofiber. (**A**) A single PLLA fiber immediately after electrospinning with uniformly distributed 6AN. (**B**) A single PLLA fiber after solvent removal shuttled 6AN from inaccessible fiber core towards the fiber surface [120]. © Elsevier Ltd. All rights reserved 2017.

Theoretically, the solvent needs to volatilize in the electrospinning process, but there is always a little solvent remains in the fiber. As an effective strategy, thermal treatments could promote the volatilization of residual solvent. D’Amato et al. [120] have developed a new method for removing residual solvents to prolong the release time of small molecules in electrospun fibers (Figure 9). Firstly, degradable poly(L-lactic acid) (PLLA) composite nanofibers containing hydrophobic drug 6-aminonicotinamide (6AN) were prepared by using electrospinning technology. Then the composite nanofibers were placed in laboratory environment for 28 days, and heat-treated at fixed temperature and environment in incubator that was maintained at 37 °C, 5% CO_2_, and 90–95% relative humidity. Compared with the untreated blank sample releasing 6AN over 9 days, the drug release time of PLLA composite nanofibers with heat-treatment can be extended to over 44 days.

#### 2.4.2. Surface Modifications

The surface properties of nanofibers play an important role in packaging materials. Although electrospinning technology can produce fiber membranes with special internal structure, the surface properties of fiber membranes still cannot meet the application in food packaging fields, such as wettability, adsorption, etc. Thus, the subsequent modification is needed to change or modify surface of electrospun nanofibers in order to meet the packaging field characteristics and special application requirements [121]. There are two usual strategies to modify the surface of electrospun film, one is grafting on the surface and the other is plasma treatment on the surface.

In the field of food packaging, the surface modifications for electrospun fiber membrane are often used to endow the membrane with antibacterial properties. For example, Li et al. [122] prepared the surface of electrospun poly(D,L-lactide) (PDLLA) membrane by plasma pretreatment, UV initiated graft copolymerization of 4-vinylpyridine (4VP) and quaternization of the grafted pyridine groups with hexylbromide. The antibacterial rate of the surface modified electrospun fiber membrane against gram-positive *Staphylococcus aureus* and gram-negative *Staphylococcus* was 99.999%. 

Additionally, surface modifications can also improve the physical properties of electrospun fiber membrane, such as mechanical properties. Surucu et al. [123] reported a dielectric barrier discharge (DBD) Ar + O_2_ and Ar + N_2_ method to modify the surface of PCL/chitosan/PCL nanofibers. The plasma modifications based on Ar + O_2_ had improved the mechanical properties and oxygen functionality.

#### 2.4.3. Dip-Coating of Electrospinning Membrane

The dip coating method is to immerse the electrospun fiber membrane in a container for a period of time and take it out, so that the coating is attached to the electrospun fiber. The advantages of dip coating are high production efficiency and simple operation.

Some researchers coated electrospun fibers by the solution or suspension of antibacterial substances to make the composite fibers antibacterial properties. Ignatova et al. [124] combined electrospinning and impregnation techniques to prepare new materials of caffeic acid phenethyl ester (CAPE)/PVP-n-poly (3-hydroxybutyrate) (PHB). The prepared new CAPE-containing material has good antioxidant activity, suggesting that addition or coating of CAPE to the fiber can completely kill Gram-positive *S. aureus* and inhibit the growth of Gram-negative *E. coli*, which is expected to be used in the field of antimicrobial packaging. 

Goha et al. [125] prepared beadless, smooth surface PLA/CS nanofibers by electrospinning, and then coated PLA nanofibers with cerium-doped bioactive glass (CeBG), copper-doped bioactive glass (CuBG) and silver-doped bioactive glass (AgBG). Then they tested the bacteriostatic activity against *Escherichia coli* (ATCC 25,922 strains) by the disk diffusion method, and found that the CeBG and CuBG modified PLA/CS nanofibers did not produce bacteriostatic areas against *E. coli* in three samples.

Yakub et al. [126] also combined electrospinning technology with the impregnation method to prepare PCL/CS composite nanofiber by using the natural phenolic acid ferulic acid (FA) as raw materials. The experimental results showed that a combination of FA and CS in the fibers is more effective for killing *Staphylococcus aureus* than FA-containing mats or CS-coated mats, and all the composite fibers containing CS and FA have a good antimicrobial activity.

This method can also modify the electrospun fiber membranes from hydrophobicity to hydrophilicity. Hu et al. [127] fabricated PLA/beta-tricalcium phosphate (b-TCP) composite fiber membranes, after that, the membrane was immersed in 5.0% (w/v) polyethylene oxide solution at room temperature for 5 minutes. After the dried, hot pressed and other steps, the surface modified composite fiber membranes were obtained, which exhibited an enhanced degradation rate, and a change of surface characteristic from hydrophobic to hydrophilic. 

## 3. Functional Materials for Food Packaging Applications 

The purpose of food packaging is to ensure the quality and safety of food, provide convenience for users, highlight the appearance and mark of commodity packaging and improve the value of merchandise. Most importantly, food packaging can prevent food deterioration and ensure food quality. With the progress of science and technology and the improvement of people’s consumption level, functional food packaging is more and more crucial in people’s daily life.

Fresh keeping, environmental protection and convenience are the new development direction of food packaging. Choosing proper packaging materials should not only consider the requirements of consumers and the needs of producers, but also consider the coordination and influence with the environment. In principle, the electrospun nanofibers used in food packaging must have biocompatibility and low toxicity, even non-toxic. Based on these above considerations, functional food packaging materials gradually require some special characteristics like degradable, super-hydrophobic, self-cleaning, edible, antibacterial and high barrier. 

### 3.1. Degradable Electrospun Packaging Membrane

At present, most of the packaging materials are polymers, such as polyethylene, polypropylene, polystyrene, polyvinyl chloride and so on, which are very stable in nature and difficult to degrade. The employment of these materials in packaging industry has caused a serious white pollution due to numerous consumptions of disposable goods, such as disposable tableware and plastic bags. Therefore, in the field of functional packaging, many scholars began to study polymers that could be able to degrade in the natural environment.

The degradable packaging materials generally refer to degradable plastics. According to the traditional classification, degradable plastics can be divided into two categories: photodegradable plastics and biodegradable plastics. So far, biodegradable polymers are very popular in the functional packaging field. Some chemically synthesized polymers, such as PCL, PPC, PLA and PVA are often used in food packaging materials through electrospinning. The presence of these polymers with good biocompatibility can provide a basic comprehension for functional modifications of packaging materials.

Biodegradable polymer materials synthesized by chemical method are similar to natural polymers or polymers with easily degradable functional groups, which can be designed and adjusted to meet the actual needs.

PCL possesses high biocompatibility and biodegradability, which always is applied as degradable packaging. Katsube et al. [128] prepared electrospun PCL nanofibers by using a solution of 12 wt. % PCL in acetone with a capillary flow rate of 24 mL/h, and an electric field of 1.005 kV/cm. Furthermore, the mechanical behavior of electrospun PCL under tensile loading was investigated. Subramanian et al. [129] studied the formation of nanofibers during electrospinning process by using melting PCL as spinning solution, and explored the effects of process parameters such as applied voltage, electrode spacing and molecular weight on fibers diameter. In this work, the diameter distribution was 5–20 microns. By controlling process parameters, the proportion of fine fibers in melt-electrospun PCL mesh was increased. Gaudio et al. [130] used 14% w/v solutions in mixture 1:1 of tetrahydrofuran and N, N-dimethylformamide and chloroform to fabricate micrometric and submicrometric fibrous PCL. The experiments have proved that PCL was nontoxic. Additionally, Bhullar et al. [131] used environmentally friendly and non-toxic melt spinning to obtain PCL micro-fibrous structure, followed by impregnation of bioactive rosemary extract. As expected, the rosemary extract was uniformly dispersed in the PCL microfiber structure. Finally, the bioactive packaging materials with satisfactory antimicrobial, structural and thermal properties can be obtained. The active bio-composite is biodegradable and biocompatible, which is able to replace traditional packaging materials.

CO_2_ can be copolymerized with epoxy monomers to obtain a series of biodegradable polycarbonates, among which PPC is an alternating copolymer of carbon dioxide and propylene oxide (PO). Using CO_2_ as monomer to synthesize PPC can not only overcome the shortage of petroleum resources, but also help to reduce carbon dioxide pollution. Generally speaking, PPC has good tensile toughness, transparency, biocompatibility and biodegradability. Park et al. [132] used sol–gel electrospinning to prepare pure PPC nanofibers, and found that the mechanical properties of pure PPC were significantly improved after the heat treatment at 60 °C due to the highly bonded structure of nanofibers, which was further interpreted by SEM diagram. Nagiah et al. [133] obtained PPC ultrathin fibers with 10% w/v polymer solution, which have good thermal stability, mechanical properties and high porosity.

PLA is defined as the most promising new packaging material in the new century by the industry, which has a series of advantages such as complete biodegradation, environmental friendliness and recyclability. PLA can be made into film products with high transparency, excellent processability and mechanical properties, which are often used in food packaging. Li et al. [134] studied the morphology, structure and tensile properties of electrospun PLA porous nanofibers with different crystallinity. It was reported that rigid porous PLA nanofibers with high tensile modulus, high strength and small strain can be obtained by the denser structure and enhanced molecular orientation during electrospinning. The study from Casasola et al. [135] demonstrated that the effects of solution properties on the morphology and diameter of PLA nanofibers. It was found that there was the highest fiber productivity in the solvent system of acetone/dimethylformamide, which could prepare the defect-free nanofibers. In this system, the effect of polymer concentration on the formation of PLA nanofibers was also studied. The structure of PLA nanofibers obtained from the critical chain entanglement concentration (CE) was beaded and the defect-free nanofibers were obtained by increasing the concentration about twice higher than that of the entanglement concentration. Critical chain entanglement concentration has positive correlation with its elastic modulus and plastic modulus. In addition, the higher the conductivity of the solvent, the finer the diameter of the fiber.

**Figure 10:**
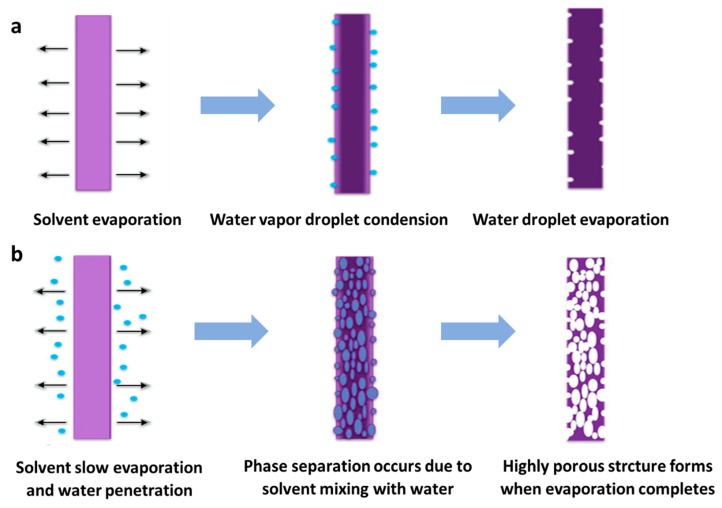
(**a**) Schematic diagram of surface pore formation induced by breath figures mechanism. (**b**) Schematic diagram of porosity induced by a vapor induced phase separation (VIPS) mechanism [137]. © Elsevier Ltd. All rights reserved 2018.

Tang et al. [136] obtained a kind of electrospun PLA nanofibers with remarkable nano-porous surface and super high specific surface area. The formation mechanism of nanopores on the surface of fibers is solvent phase separation. Huang et al. [137] further studied the effect of different solvents on the surface morphology and internal porous structure of electrospun PLA fibers. Both surface and internal pore can be achieved through different mechanisms. It could be clearly found in Figure 10 that by choosing different solvent systems, electrospun PLA nanofibers with porous surface or inner surface and their combination can be obtained under suitable humidity and environment conditions.

Tomaszewski et al. [138] studied the effects of molecular weight of PLA and spinning solution viscosity on the thickness and quality of fiber felt. In addition, the thermal and tensile properties of fiber felt were also studied. Li et al. [139] came to a similar but more detailed conclusion, which are the uniform fibers that can only be obtained when the concentration of low molecular weight PLA solution is above the entanglement concentration. On the contrary, when the concentration of high molecular weight PLA solution is below entanglement concentration, uniform fibers cannot be obtained. Meanwhile, due to the low viscosity and deformation resistance of precursor solution, the PLA nanofiber has remarkable molecular alignment, which also leads to a rapid cold crystallization and high modulus in the nanofibers.

**Figure 11:**
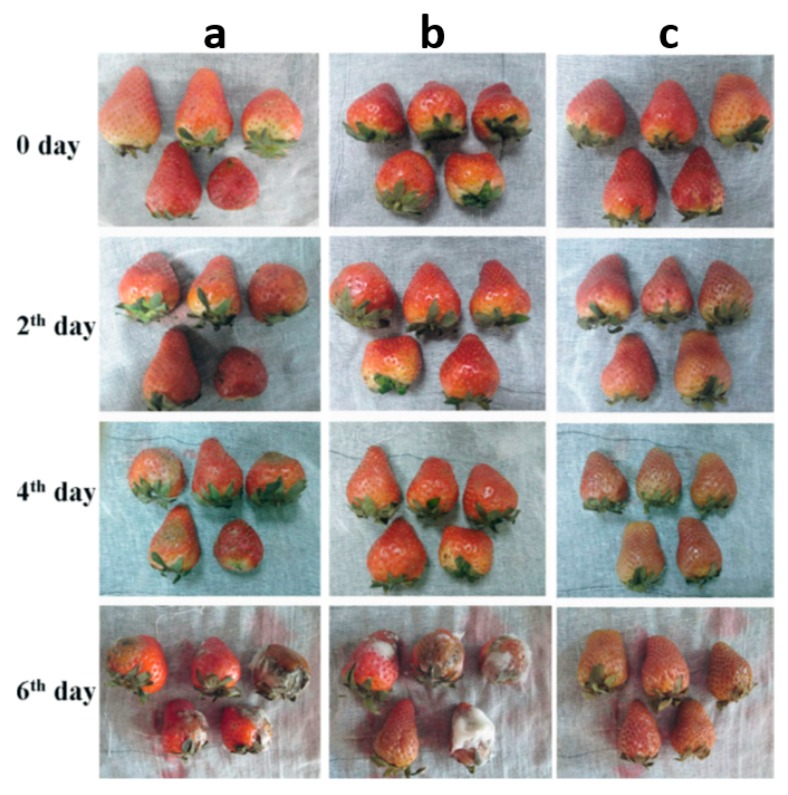
Appearance changes of strawberries stored at 21 °C. (**a**) Control; (**b**) packed with fresh-keeping film and (**c**) packed with PVA/cinnamon essential oil /b-CD nanofilm [141]. © Elsevier Ltd. All rights reserved 2015.

In the field of food packaging, biodegradable PVA is often combined with other substances to achieve the target functionality owing to some simple electrospinning conditions. Kayaci et al. [140] successfully produced PVA nanowebs incorporating vanillin/cyclodextrin inclusion complex (vanillin/CD–IC) via an electrospinning technique with the goal to obtain functional nanowebs containing flavor/fragrance molecules with enhanced thermal stability and durability. Therefore, PVA/vanillin/CD–IC nanowebs can be quite applicable in active food packaging. Wen et al. [141] fabricated electrospun PVA/cinnamon essential oil/b-cyclodextrin (PVA/CEO/b-CD) antimicrobial nanofibrous film, which can effectively prolong the shelf-life of strawberry, indicating it is potential for the application in active food packaging (Figure 11).

### 3.2. Superhydrophobic Electrospun Packaging Membrane

The superhydrophobic surface has the advantages of self-cleaning and anti-adhesion, which could delay the deterioration of food and prevent the propagation of microorganisms in food packaging, so the superhydrophobic packaging membrane is very popular in this field.

Ding et al. [142] obtained superhydrophobic fibrous PVA/ZnO composite films by electrospinning and surface treatment with fluoroalkylsilane (FAS). First, ZnO with nanostructured surface was formed by calcining the electrospun composite nanofiber membranes, and then the surface was modified by fluoroalkylsilane coating to obtain the superhydrophobic surface, and the contact angle of the rest with water has changed from 0 to 165 degrees, which means that the surface of composite was also changed from superhydrophilic to superhydrophobic. The comparative experiments showed that the superhydrophobic surface of the composite fiber membrane is the result of combination of high surface roughness and hydrophobic FAS modification. 

**Figure 12:**
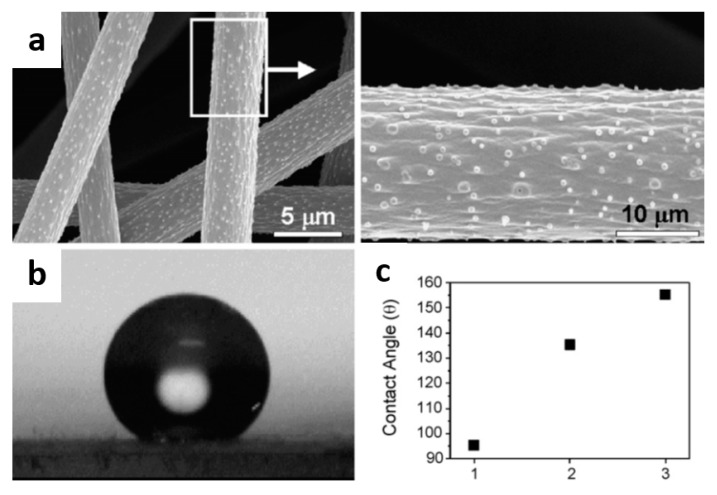
(**a**) FESEM images of electrospun PS fibers from 35 wt.% solution in DMF, (**b**) water droplet on electrospun PS fibers from 35 wt.% solution in DMF and (**c**) variation of water contact angles depending on surface structures (1: PS film; 2: electrospun PS fibers using THF; 3: electrospun PS fibers from DMF) [144]. © Elsevier B.V. All rights reserved 2007.

Actually, the rough surface has played an important role on the superhydrophobic surface, which involves a principle denoted as lotus leaf effect that is an important part for the superhydrophobic surface because there are many micron scale protrusions on the lotus leaf surface. The rough structure increases the air fraction in the space–time contact between the surface and water, and greatly reduces the actual contact area between water and lotus leaf [143]. Therefore, many research groups use the so-called nano plate making technology to prepare the surface with artificial lotus leaf. For instance, Kang et al. [144] utilized the volatility of solvent during electrospinning to prepare PS films with unique protuberances, which the structure is similar to the surface of the lotus leaf, so the electrospun fiber membrane has a superhydrophobic property, the water contact angle is 154.2 ± 0.7° (Figure 12).

### 3.3. Edible Electrospun Packaging Membrane

The application of edible packaging in food packaging has a long history. For decades, the familiar glutinous rice paper used in candy packaging and the corn baking packaging cup used in ice cream packaging were typical edible packaging. With the improvement of people’s requirements for food quality and preservation period, as well as the enhancement of people’s awareness of environmental protection, edible films consisted of natural biological materials are becoming a research hotspot in the field of food packaging. Generally, edible packaging membranes have different kinds of ingredients according to their compositions. The first one is polysaccharide membranes, such as starch, cellulose derivative, pectin, chitosan, etc. the second one is protein membranes, such as collagen, gel, and the third one is lipid membranes, such as beeswax, paraffin, the fourth kinds is compound membranes, which are obtained through the combination of three above substances.

**Figure 13:**
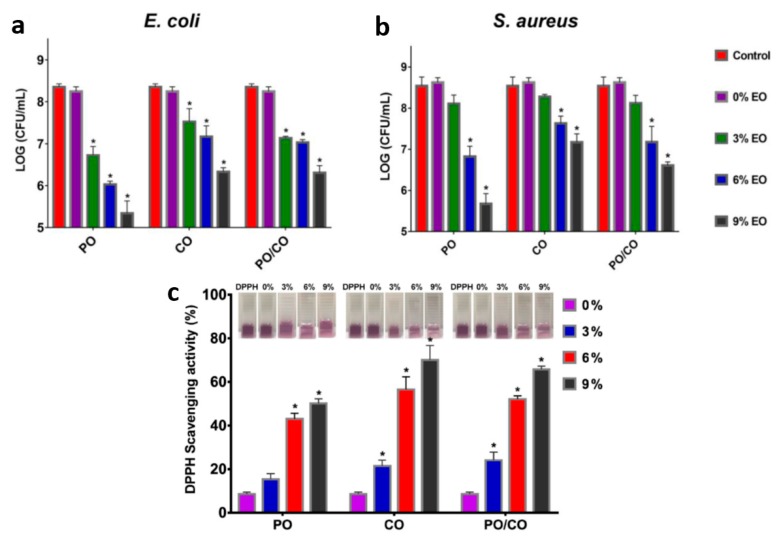
(**a**,**b**) The results of dynamic contact assays against *Escherichia coli* and *Staphylococcus aureus.* (**c**) The antioxidant performance of gelatin/EOs nanofibers was determined using the DPPH radical scavenging method, (∗) *p* (in Tukey’s post hoc test) < 0.05 versus the control group [145]. © American Chemical Society 2019.

Tang et al. [145] prepared edible gelatin based composite fibers by electrospinning. The combination of gelatin nanofibers with peppermint essential oil (PO) and chamomile essential oil (CO) could enhance the hydrophobicity of membrane surface, and all the gelatin nanofibers containing PO, CO or PO/CO had better antibacterial properties against *Escherichia coli* and *Staphylococcus aureus*, and had certain antioxidant properties (Figure 13). In particular, the addition of PO leads to a better antibacterial activity of the fiber membrane, while the oxidation resistance of fiber membrane containing CO is better.

Based on one-step electrospinning technology, Mascheronia et al. [146] proposed an edible polysaccharide system that can be applied to food packaging. In this system, the cyclodextrin crystal is surrounded by aromatic compounds and fixed on the Prussian nanofiber mesh. The composite, combined with the membrane’s pullulan nanofibers, is stable for several months without loss of volatiles when it was stored in ambient relative humidity and at different temperatures. Due to the ability of encapsulation and release of antibacterial aromatic compounds, the system has potential applicability for improving microbial safety, especially for fresh food.

### 3.4. Antibacterial Electrospun Packaging Membrane

According to the previous description, there are three kinds of antibacterial substances, including natural biological antibacterial agents (essential oil, etc.), organic chemical antibacterial agents (organic acid, etc.) and inorganic antibacterial agents.

Cristina et al. [147] successfully encapsulated a naturally occurring antimicrobial compound, allyl isothiocyanate (AITC) into soy protein isolate (SPI) and PLA fibers by electrospinning technology, and studied its effect on the fiber properties. By elaborately manipulating the formulation of solution, the morphology of composite nanofibers can be adjusted. Most of all, it was found that AITC released from SPI and PLA electrospun fibers could be controlled by changing relative humidity, and the increase of relative humidity of air can triggers the release of AITC in fibers.

Neo et al. [148] evaluated the applicability of gallic acid loaded zein (Ze-GA) electrospun fiber mats towards potential active food packaging. As expected, the Ze-GA fiber mats demonstrated outstanding antibacterial activity and properties consistent with those considered desirable for active packaging material in the food industry.

**Figure 14:**
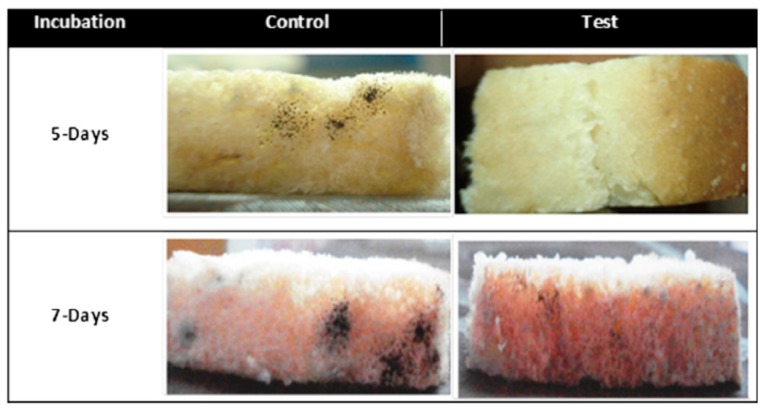
Effect of MMT-N6 nanofibrous membrane coating on PP packets on the natural flora of bread [149]. © Elsevier Ltd. All rights reserved 2014.

As a natural mineral, montmorillonite (MMT) can also be applied in food packaging industry. Agarwal et al. [149] coated PP membranes with MMT and nylon 6 nanofibers by electrospinning. The effect of these membranes in the packaging of popular foods such as crisps and bread were investigated (Figure 14). When the membranes were used in the packaging of potato chips, the oxygen barrier property was significantly reduced, which was attributed to the MMT-N6 coating that slightly improves the water vapor barrier. The reason for decrease in microbial spoilage of bread was similar. Therefore, the coated MMT composite membranes can be applied in the packaging industry due to its non-toxic, which can realize the extension of shelf life of packed food.

**Figure 15:**
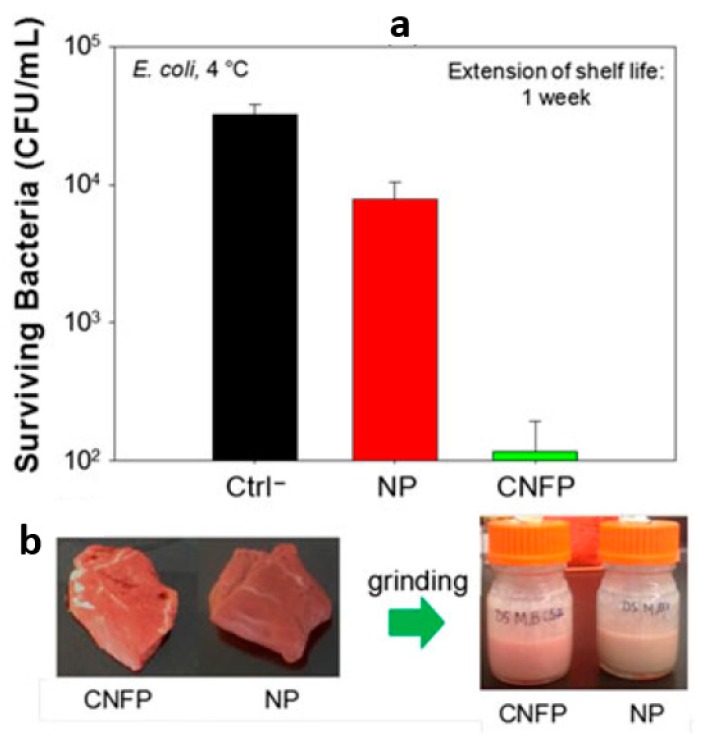
(**a**) In situ antibacterial activity of CNFP against *E. coli* after 7-day storage at 4 °C. (**b**) Appearance of packed red meat with and without CNFP, before and after grinding [150]. © John Wiley & Sons, Ltd 2018.

As mentioned above, chitosan is a natural antibacterial polymer, so it has attracted numerous attentions for the food packaging industry. Arkoun et al. [150] firstly studied the antibacterial potential of electrospun chitosan-based nanofibers (CNF) by storing with the actual foods, and further investigated its ability to reduce spoilage and food loss. They successfully obtained highly antibacterial CNF-based packaging (CNFP) materials by direct electrospinning. It was found that an advantageous potential for antimicrobial packaging materials is that the quality and freshness of unprocessed or minimally processed and perishable foods could be perfectly preserved along with the extension of meat shelf life to 1 week (Figure 15).

### 3.5. Barrier Electrospun Packaging Membrane

The barrier property of packaging materials refers to polymer materials with certain shielding ability for small molecule gas, liquid, water vapor, fragrance and drug taste. It can effectively prevent oxygen and water vapor from seeping into the environment, keep the specific gas composition in the package, and significantly improve the shelf life. At present, in order to meet the market demands, many countries have developed multi-functional high barrier packaging materials and multi-functional packaging materials in recent years.

Fabra et al. [151] developed a fully biodegradable multi-layer system based on a high barrier adhesive sandwich of electrospun zein nanofibers between poly hydroxybutyrate-co-valerate 5% (PHBV5) and poly-(3-hydroxybutyrate) (PHB) homopolymers with low valerate content. By adding zein nanofibers, the water and oxygen barrier in the multi-layer system of PHA was improved, and the flexibility was also enhanced. After that, electrospinning technology was employed to improve the barrier performance of packaging materials [152]. The effects of PCL, PHA and PLA on the oxygen and water resistance of TPCS membrane were compared. The result revealed that the PHB was the most effective in reducing the water and oxygen permeability.

The previous chapter has shown that zein can be prepared as an edible packaging film. In recent years, many scholars have also used electrospun zein film as the interlayer to enhance the barrier properties of food packaging. Fabra et al. [153] employed the electrospun zein as the middle layer and cast PHBV12 as the outer layer to prepare a multilayer structure food packaging films, which can significantly improve the oxygen barrier performance. They also compared the enhancement of the oxygen and water barrier properties of multi-layer packaging films with PHBV3 as the outer layers and different electrospun hydrocolloid films as the interlayers [153]. The result showed that oxygen and water vapor permeability values were significantly improved by employing electrospun zein film as the interlayer.

## 4. Conclusions and Challenges

In summary, the electrospinning technology is an effective method to prepare nanofibers with various nanostructure and surface characteristics, which can meet the functional requirements for packaging materials in the food fields depends on the different design of device and wide range of raw materials for selection, especially polymers. This article reviews the potential use of electrospinning technology in the food packaging field, not only to prepare pure degradable polymer packaging membranes, but also to obtain mixed polymer mats by designing the spinneret, such as multiple-jet and coaxial electrospinning. In addition, different inorganic fillers and other bioactive particles can be incorporated into the fibers to enhance the functionality and intend to be used in broader applications. The properties of common polymers and fillers mentioned above are summarized in Table 1. Alternatively, the resulting electrospun films are post-treated by the thermal treatments, surface modifications or dip-coating to obtain superior satisfactory performance. In the field of food packaging, based on some special requirements, it is possible to prepare packaging materials that own functions of degradable, superhydrophobic, edible, antibacterial and high barrier by electrospinning technology.

Although the application of electrospinning technology in functional packaging has a wide range of development prospects, there are still some challenges that need to be faced and require more attention and studies. The biggest challenge is the choice of solvent. Some polymers are insoluble in non-toxic solvents, which would cause environmental damage and pollution. Furthermore, some organic solvents are harmful to human beings. Therefore, it is necessary to find more suitable and non-toxic solvents to prepare electrospun fibers, especially green electrospinning, a novel and aqueous strategy to overcome the disadvantages of organic solvents. Up to now, some commercially available polymers in packaging industry can only be dissolved in toxic solvents. Therefore, water or other non-toxic solvents with environment friendly property would be applied to prepare food packaging films in the future. In fact, during the electrospinning process, the solvents are mostly evaporated. Unfortunately, the above organic solvents are toxic and harmful to the environment, so it is crucial to replace the conventional toxic solvents with non-toxic or low-toxic solvents, such as emulsion electrospinning method [154,155], that will minimize the presence of toxic solvents in the final packaging material. Another challenge is some natural polymers cannot be directly electrospun into nanofibers, so it is crucial to select a suitable polymer, which is also a problem of electrospun material in food packaging. Most of all, the majority of research is still in the laboratories. So, as for the real food packaging industry, it is important to produce electrospun fibers on a large scale, and the subsequent development must be focused on high yields and industrialization. Ordinary multi-nozzle electrospinning or needless electrospinning is suitable for industrial production. There are already some manufacturers that can provide large-scale devices for producing electrospinning fibers or membranes. For example, Elmarco has developed needleless electrospinning device, which they claim is suitable for industrial applications (https://www.elmarco.com). Some factories already have begun large-scale production of nanofiber membranes, such as Jiangxi Xiancai Nanofibers Technology Co., Ltd. in China for the large scale production of electrospun polyimide fibrous membranes, yarns and short nanofibers (http://www.hinanofiber.com/english/). All of these have laid some foundation for the future electrospinning production of food packaging materials, and the focus of electrospinning food packaging materials also need to be transferred from laboratory research to industrial production in the future.

## Figures and Tables

**Table 1:** Summary of promising properties related to packaging using electrospinning.

Categories	Materials	Properties or Function
Polymer	Chitosan (CS)	biodegradation, biocompatibility, anti-microbial, antifungal activities, and non-toxicity.
	Zein	good film-forming property, biocompatibility, biodegradation, renewable, edible
	Polyvinyl alcohol (PVA)	transparency, gantistatic property, biodegradation, biocompatibility
	Gelatin (GT)	biodegradation, biocompatibility, edible, good toughness
	Polycaprolactone (PCL)	biocompatibility, biodegradability, good mechanical properties, better solvent solubility
	Polyethylene terephthalate (PET)	non-toxic, good mechanical properties, high transparency, good toughness
	Cellulose acetate (CA)	non-toxic, biodegradable, low price, good transparency, high impact resistance
	Polylactic acid (PLA)	biodegradation, biocompatibility, easy to process, good mechanical properties and transparency
	Poly (propylene carbonate) (PPC)	good tensile toughness, transparency, biocompatibility and biodegradability
	Polystyrene (PS)	High transparency, non-toxic, easy to process
Inorganic fillers	Metronidazole (MNA)	hydrophobic, antibacterial
	Carbon nanotubes (CNTs)	conductive, antistatic, smart packaging
	FePt, Fe_3_O_4_, FeCl_3_ nanoparticles	radiation protection
	TiO_2_	photocatalytic, self-cleaning, photocatalytic degradation of ethylene
	ZnO	photocatalytic, self-cleaning, antibacterial
	Cerium-doped bioactive glass (CeBG), copper-doped bioactive glass (CuBG), silver-doped bioactive glass (AgBG)	antibacterial
	Montmorillonite (MMT)	antibacterial
Active substance	Orange essential oil (OEO)	antibacterial
	Metronidazole (MNA)	antibacterial
	Peppermint essential oil (PO), chamomile essential oil (CO)	antibacterial
	Vanillin/cyclodextrin inclusion complex (vanillin/CD-IC)	containing flavor/fragrance, enhancing thermal stability and durability
	Cinnamon essential oil/b-cyclodextrin (PVA/CEO/b-CD)	prolonging the shelf-life
